# Radiological Diagnosis of Crouzon Syndrome: A Case Study

**DOI:** 10.7759/cureus.62564

**Published:** 2024-06-17

**Authors:** Sudhanshu Tonpe, Himandri Warbhe, Pankaj Banode, Suhas Kommuru, Vadlamudi Nagendra

**Affiliations:** 1 Department of Interventional Radiology, Jawaharlal Nehru Medical College, Datta Meghe Institute of Higher Education and Research, Wardha, IND; 2 Department of Respiratory Medicine, Jawaharlal Nehru Medical College, Datta Meghe Institute of Higher Education and Research, Wardha, IND; 3 Department of Radiology, Government Medical College, Nirmal, Nirmal, IND; 4 Department of Radiology, Jawaharlal Nehru Medical College, Datta Meghe Institute of Higher Education and Research, Wardha, IND

**Keywords:** 3d c, exophthalmos, copper beaten appearance, crouzon syndrome, craniofacial dysostosis

## Abstract

Crouzon syndrome, distinguished by a classic trio of an atypical skull structure, distinctive facial features, and protruding eyes, ranks among the most prevalent types of craniofacial dysostosis. Therefore, patients presenting with dental abnormalities are under-reported in medical literature despite the developmental neurological defects. We report a rare case of Crouzon syndrome in a four-year-old girl who had forward displacement of the lower jaw, bulging eyes, undeveloped upper jaw, and dental abnormalities. She was evaluated with cranial computed tomography with three-dimensional reconstruction; genetic studies confirmed the findings.

## Introduction

Crouzon syndrome is an uncommon genetic disorder identified by a combination of skull malformations, typically the cause of early closure of cranial sutures known as craniosynostosis, underdevelopment of the midface, and ocular irregularities commonly presenting as exophthalmos and dental abnormalities [1-5]. A mutation in the fibroblast growth factor receptor two (FGFR2) gene is thought to be the cause of Crouzon syndrome. It represents around 4.8% of all cases of craniosynostosis, making it the most prevalent syndrome within this group.

Its global prevalence is estimated to be around 1 per 25,000 live births [5-7]. It differs from other craniosynostosis syndromes in which the hands and feet do not exhibit any abnormalities [5]. Timely clinical and radiological diagnosis plays a pivotal role in averting the progression of debilitating conditions such as mental retardation, visual impairment, hearing loss, and airway obstruction in individuals with Crouzon syndrome [5,8,9].

## Case presentation

**Figure 1:**
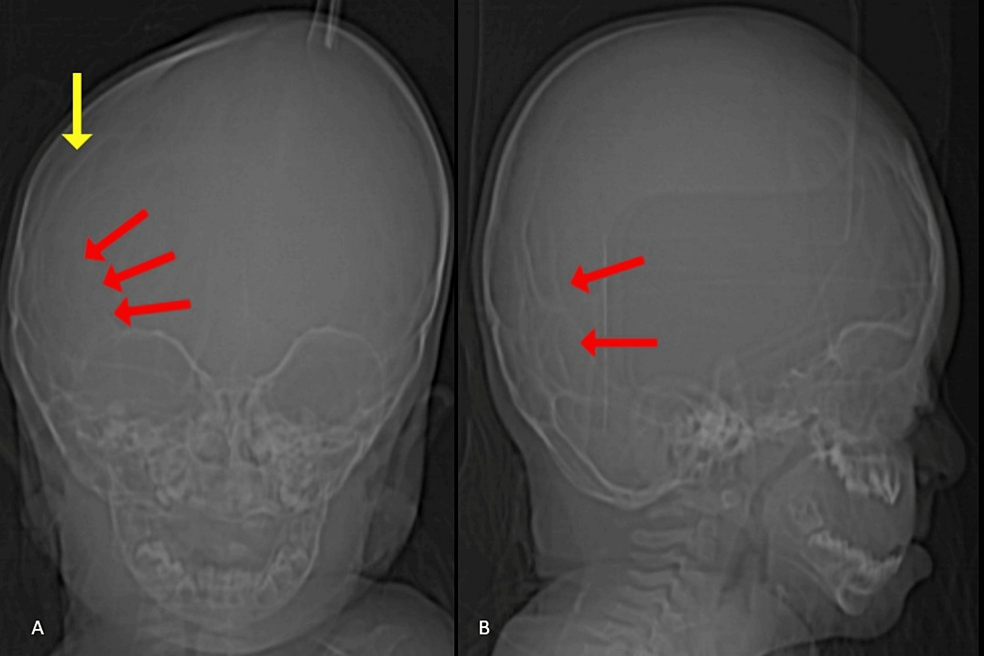
Anterior-posterior (A) and lateral (B) radiographs of the skull demonstrating copper-beaten appearance (red arrow) due to raised intracranial pressure and fusion of the right coronal sutures (yellow arrow).

A four-year-old girl presented to the hospital with dental abnormalities, exophthalmos, maxillary hypoplasia, and mandibular prognathism. Siblings, family, and near relatives showed no anomalies and medical history. The mother reported that she had a normal labor and delivery and noticed an enlarged skull from the time of birth. There was no abnormality in the limbs. The skull radiograph showed a copper-beaten appearance due to raised intracranial pressure and fusion of the right coronal sutures (Figure 1).

**Figure 2:**
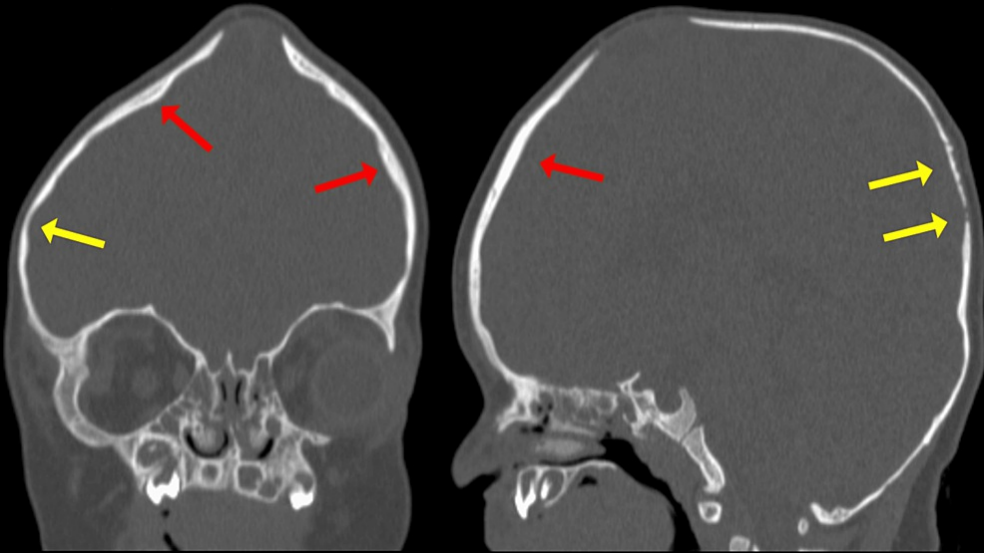
The head computed tomography (CT) scan revealed regions where skull bones varied in thickness (thick – red arrow, thin – yellow arrow).

**Figure 3:**
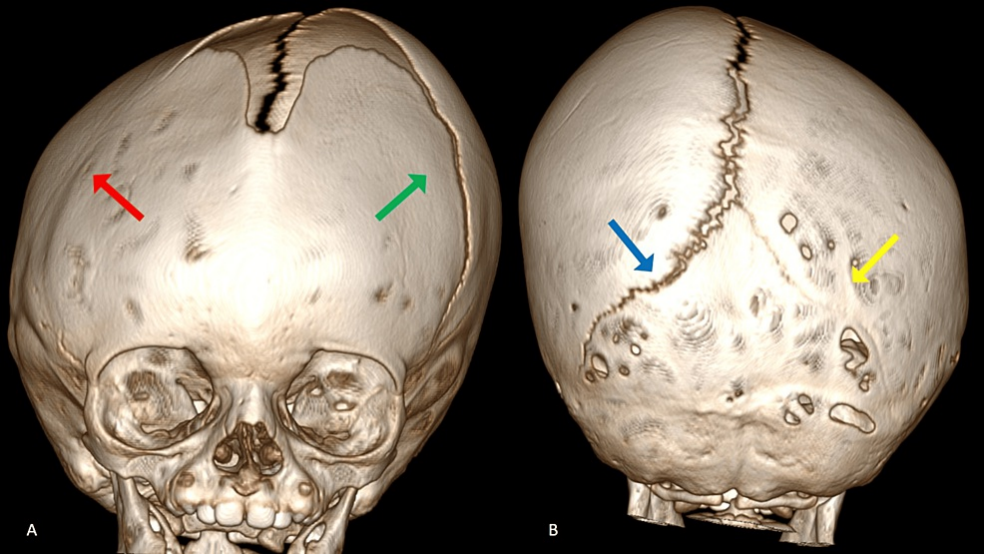
3D reconstruction of the skull. The fused bony junctions on the right coronal (red arrow) and left lambdoid sutures (yellow arrow) of the head, whereas the seams on the opposite sides, specifically the left coronal (green arrow) and right lambdoid (blue arrow), appeared normal.

The head computed tomography (CT) scan revealed regions where skull bones varied in thickness. Moreover, the bony junctions on the right top and left back of the head, known as the coronal and lambdoid sutures respectively, were fully fused, whereas the seams on the opposite sides, specifically the left coronal and right lambdoid, appeared normal (Figure 2). Brain parenchyma was normal. The CT results were validated using three-dimensional (3D) reconstruction (Figure 3).

Sanger sequencing ABI PRISM® genetic tests revealed a heterozygous mutation in Exon 8 (c. 1025 > A, p. Cys342Tyr) of the Chromosome 10 FGFR2.

The patient was advised surgery; however, consent was not provided and the patient has delayed developmental milestones after one year of age. 

## Discussion

Crouzon syndrome is an uncommon disorder that affects around 1 in 25,000 live births globally and accounts for 4.8% of all cases of craniosynostosis. In 1912, Louis Edouard Octave Crouzon, a French neurosurgeon, initially described it as a hereditary craniosynostosis syndrome featuring skull deformities, facial anomalies, and exophthalmos. Moreover, correlations with bifid uvula, cleft lip, and cleft palate are identified. This disorder has varying expressivity but complete penetrance in an autosomal dominant manner. Anomalous molding of the skull and brain occurs perpendicular to the fused sutures, resulting in craniofacial deformities [1-3,8-12].

Premature craniosynostosis, absence of digital and limb anomalies, and craniofacial anomalies are the radiographic hallmarks of Crouzon syndrome. The coronal, lambdoid, or sagittal sutures may be affected by premature craniosynostosis, and it may even encompass the sutures at the base of the skull, resulting in mid-face hypoplasia and obstruction of the upper airways. Furthermore, there may be spinal malformations such as fused cervical vertebrae, butterfly vertebrae, and anomalies at the craniovertebral junction.

Radiographs of the anterior, posterior, and lateral skulls show suture fusion, sclerosis, and uneven form. Sutural fusion can manifest as either partial or complete suture obliteration, resulting in fused sutures displaying a "heaped up" appearance due to the absence of normal interdigitations. Additional radiographic features of craniofacial bones in illnesses like Crouzon syndrome include a hammered silver beaten/copper beaten appearance of the skull vault, shallow orbits, an inflated hypophyseal cavity, hypoplastic maxilla, and small paranasal sinuses [1,4,13,14].

The width of the sagittal suture is estimated to be 5 mm and that of the coronal suture is 2.5 mm at birth. These measurements gradually decrease to around 1.5 mm and 0.8 mm, respectively, by the age of one year, eventually leading to fusion between the ages of 40 and 60 years. In individuals with craniosynostosis, the sutures are not visible or discernible on radiographic imaging. Prematurely fused sutures can affect symmetry, either symmetrically or asymmetrically [15,16].

Few studies have highlighted the importance of cranial CT, especially with 3D reconstruction, in determining the parasutural bony ridges in individuals with craniosynostosis, diagnosing parasutural sclerosis, and evaluating the state of specific skull sutures. CT scans are also crucial for evaluating the outcomes of surgical interventions. While magnetic resonance imaging is not typically performed as part of routine assessment, it may prove beneficial in syndromic cases for detecting brain parenchymal abnormalities that may have been missed on CT scans [13,16,17].

Apert syndrome, Jackson-Weiss syndrome, Carpenter syndrome, Pfeiffer syndrome, Saethre-Chotzen syndrome, and Crouzon syndrome with acanthosis nigricans are among the illnesses included in the differential diagnosis of Crouzon syndrome [4,5,13,18-21]. Unlike Crouzon syndrome, these syndromes typically exhibit digital and limb abnormalities.

The first year of life is typically when Crouzon syndrome treatment starts, and it involves frontal-orbital advancement and cranial decompression. These measures are intended to prevent elevated intracranial pressure, which can result in issues such as mental retardation and visual impairment [13]. All surgical approaches are geared toward augmenting cranial vault volume and alleviating elevated intracranial pressure in Crouzon syndrome. The "Ilizarov procedure," which is frequently advised for this disease, has been shown to accomplish complete exophthalmos correction and improve the midface region's esthetic look. Another surgical intervention, "distraction osteogenesis," is also utilized in the treatment of craniosynostosis. Few studies have highlighted the importance of cranial CT, especially with 3D reconstruction, in determining the parasutural bony ridges in individuals with craniosynostosis, diagnosing parasutural sclerosis, and evaluating the state of specific skull sutures. These assessments indicate the successful post-procedural decrease in intracranial pressure [22].

## Conclusions

In conclusion, if left untreated, Crouzon syndrome can lead to adverse effects on facial aesthetics and potential complications such as airway obstruction, cognitive impairment, and declining visual acuity as the individual ages. Therefore, early management is crucial to mitigate these risks and improve overall outcomes. Cranial CT, particularly with 3D reconstruction, holds significant importance in evaluating sutures and strategically planning timely surgical interventions related to sutures. Radiological assessments not only aid in confirming diagnoses but also play a vital part in the essential postoperative evaluation of surgical outcomes. With appropriate treatment, individuals with this condition can lead fulfilling and active lives as integral members of society.
